# Rowhammer Attacks in Dynamic Random-Access Memory and Defense Methods

**DOI:** 10.3390/s24020592

**Published:** 2024-01-17

**Authors:** Dayeon Kim, Hyungdong Park, Inguk Yeo, Youn Kyu Lee, Youngmin Kim, Hyung-Min Lee, Kon-Woo Kwon

**Affiliations:** 1 Department of Computer Engineering, Hongik University, Seoul 04066, Republic of Korea; danhandev@g.hongik.ac.kr (D.K.); parkrokr@g.hongik.ac.kr (H.P.); yik6465@g.hongik.ac.kr (I.Y.); younkyul@hongik.ac.kr (Y.K.L.); 2 School of Electronic and Electrical Engineering, Hongik University, Seoul 04066, Republic of Korea; youngmin@hongik.ac.kr; 3 School of Electrical Engineering, Korea University, Seoul 02841, Republic of Korea

**Keywords:** dynamic random-access memory, rowhammer, security, vulnerability

## Abstract

This paper provides a comprehensive overview of the security vulnerability known as rowhammer in Dynamic Random-Access Memory (DRAM). While DRAM offers many desirable advantages, including low latency, high density, and cost-effectiveness, rowhammer vulnerability, first identified in 2014, poses a significant threat to computing systems. Rowhammer attacks involve repetitive access to specific DRAM rows, which can cause bit flips in neighboring rows, potentially compromising system credentials, integrity, and availability. The paper discusses the various stages of rowhammer attacks, explores existing attack techniques, and examines defense strategies. It also emphasizes the importance of understanding DRAM organization and the associated security challenges.

## 1. Introduction

Dynamic Random-Access Memory (DRAM) has gained widespread adoption as the main memory across a spectrum of computing systems, ranging from smartphones and personal computers to workstations and servers. The pervasive usage of DRAM can be attributed to its inherent advantages, such as low latency, high density, and low cost per bit. However, within the realm of DRAM’s strengths lies a security vulnerability known as *rowhammer*. This vulnerability, first introduced in 2014 [1], has been extensively researched, revealing its significant potential to compromise the confidentiality, integrity, and availability of computing systems [2,3,4,5,6,7,8,9].

A typical rowhammer attack involves the repetitive access of a specific row within a DRAM chip [1]. This aggressive access can inadvertently trigger bit flips in neighboring victim rows, causing unexpected changes in their values. Many research groups have explored various repetitive access patterns, such as single-sided, double-sided, or multi-sided access patterns, in an effort to enhance the probability of inducing bit flips or to circumvent rowhammer defense mechanisms, such as target row refresh [10,11,12,13,14,15,16,17,18].

Previous studies have emphasized the importance and challenges of a preparatory stage prior to accessing a DRAM row, which relies on a comprehension of the operating system and the underlying processor architecture. For example, the identification of DRAM row adjacency for a rowhammer attack necessitates memory profiling since the consecutive memory addresses used by a program do not align linearly with DRAM rows. Moreover, the rapid repetitive activation of a DRAM row to induce bit flips requires bypassing a cache hierarchy employed to minimize DRAM memory accesses.

**Figure 1:**
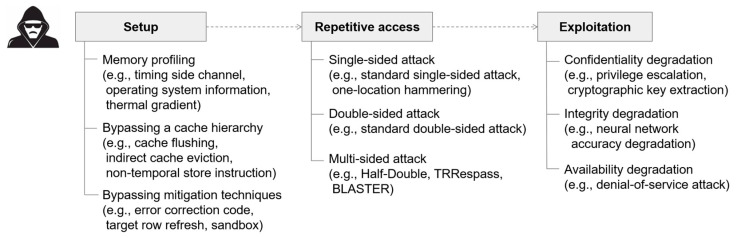
Three stages of rowhammer attack.

**Figure 2:**
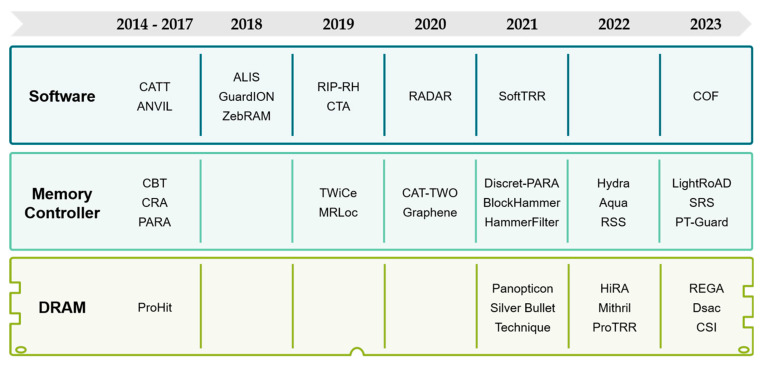
Defense mechanisms against rowhammer attacks [1,10,11,12,13,14,15,16,17,18,19,20,21,22,23,24,25,26,27,28,29,30,31,32,33,34,35,36,37,38,39,40,41,42,43].

This paper provides a comprehensive review of recent advancements in rowhammer attacks. To elucidate, we deconstruct the attack process into three stages: (1) setup, (2) repetitive access, and (3) exploitation, as illustrated in Figure 1. Subsequently, we present existing techniques within each stage, delving into their underlying mechanisms. Additionally, we conduct an exhaustive examination of defense strategies against rowhammer attacks, categorizing them into three distinct groups based on where each mitigation technique is applied, as depicted in Figure 2.

The rest of this paper is organized as follows. Section 2 provides a brief review of DRAM organization and the rowhammer mechanism. Section 3 discusses the setup stage that initiates the rowhammer attack. Section 4 reviews various row access patterns employed in executing rowhammer attacks, categorized into single-sided, double-sided, and multi-sided attacks. Section 5 examines applications of rowhammer attacks that pose threats to the credentials, integrity, and availability of computing systems. Section 6 discusses existing mitigation techniques, including software-based, memory controller-based, and DRAM-based approaches. Section 7 discusses new challenges and needs for rowhammer research on DDR5 DRAM, and Section 8 concludes the paper.

## 2. Background

### 2.1. DRAM Background

**Figure 3:**
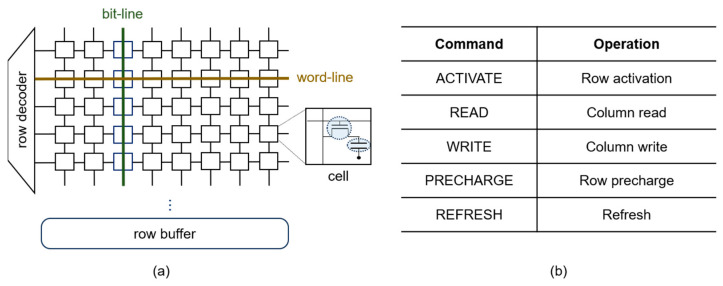
(**a**) DRAM organization and (**b**) DRAM commands.

DRAM is organized as a hierarchy of two-dimensional arrays of cells, where each cell is responsible for storing a single bit of data using a combination of a capacitor and an access transistor as shown in Figure 3a. The binary data value of each cell is determined by the charge state of its capacitor. To access and retrieve data from individual cells, *word-lines,* and *bit-lines* are employed to establish the path to the desired data location. The word-line connects to all cells in the same row horizontally, while the bit-line connects all cells in the same column vertically.

When the word-line is activated, typically by issuing a row activation command, commonly referred to as an ACTIVATE command, it enables all the access transistors within that row, establishing connections between the capacitors to their respective bit-lines (See Figure 3b). This operation transfers the data from the row into a dedicated row buffer. The row buffer reads the charge from the cells, resulting in the inevitable charge leakage in the cells, and promptly writes this charge back into the cells. Subsequently, all access operations on a subset of the row, such as a column read command (READ) or a column write command (WRITE), are managed by the row buffer on behalf of that row. When there are no further accesses to the row, the word-line voltage is de-asserted to disengage the capacitors from the bit-lines, typically accomplished by issuing a precharge command known as PRECHARGE.

DRAM poses inherent challenges in data retention due to various leakage mechanisms, including gate-induced drain leakage and subthreshold leakage [1,44,45]. To mitigate data loss, the cell’s charge must undergo restoration, a process known as refreshing. This involves activating the row to which the cell belongs. Upon row activation, the row buffer reads the cell’s charge value and promptly restores it to its original state. For more efficient refresh operations involving multiple rows simultaneously, DRAM has a dedicated command known as REFRESH [1].

### 2.2. Rowhammer Mechanism

Rowhammer is a security vulnerability in DRAM, enabling an attacker to change the data stored within memory cells. Rowhammer attacks take advantage of the physical proximity of these cells within the memory array. By repetitively and rapidly activating a specific row, it can result in charge leakage from the capacitors of victim cells in adjacent rows [46,47,48,49]. Three potential causes for this phenomenon have been hypothesized, as posited by [47]:

(1) Bridging between neighboring rows: The study in [50] demonstrated the formation of conductive channels between separate wires and capacitors in DRAM. Additionally, the study in [48] illustrates that the frequent toggling of a word-line can expedite the charge flow between two bridged cells.
(2) Electromagnetic coupling: The alteration of voltage in a word-line can introduce noise into a neighboring word-line via electromagnetic coupling, inducing the leakage of charge from the victim cells [49,51,52].
(3) Hot carrier injection: Prolonged toggling of a word-line can lead to hot-carrier injection [53]. The injection of hot carriers into adjacent rows may escalate charge leakage from victim cells.

**Figure 4:**
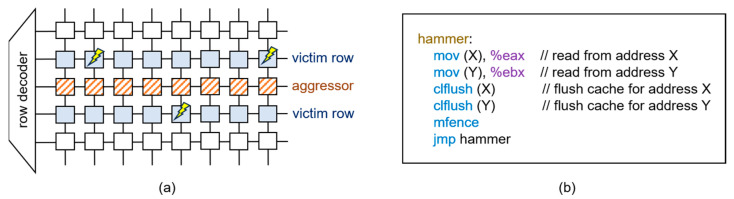
(**a**) Illustrative example of bit flips and (**b**) x86 assembly code for rowhammer attack.

This charge leakage may cause some victim cells to fail to retain their charge for the prescribed refresh interval. Consequently, this phenomenon leads to the alteration of the stored data, resulting in a bit flip from 0 to 1 or vice versa, as illustrated in Figure 4a.

Rowhammer attacks represent a significant security concern because they bypass traditional security mechanisms and can be conducted using software applications without requiring physical access to the targeted machine. Figure 4b shows x86 assembly code that induces the rowhammer effect, assuming that memory addresses X and Y are mapped to different DRAM rows in the same memory bank [1].

## 3. Setup for Rowhammer

This section discusses the preparation steps leading up to initiating a rowhammer attack. It begins with an examination of memory profiling, followed by techniques for circumventing the cache hierarchy to gain direct access to DRAM cells. Lastly, it explores strategies for overcoming different defenses against rowhammer attacks. Table 1 provides a summary of the referenced papers that pertain to the setup of a rowhammer attack.

### 3.1. Memory Profiling

In order to carry out a successful rowhammer attack, the attacker must repeatedly access a specific row that is adjacent to the row containing victim cells. This operation requires an understanding of DRAM address mapping since, typically, software and processors employ virtual addresses and physical addresses, respectively, rather than DRAM addresses.

One possible method for establishing virtual-to-physical address mapping involves accessing information provided by the operating system. For example, in Linux, the/*proc/self/pagemap* file contains comprehensive data regarding the translation from virtual to physical addresses. Given that this file was initially accessible to users, prior research conducted by [2,55,60] has demonstrated that processes in userland can gain knowledge of the physical memory layout. (The Linux kernel has discontinued unprivileged access to the/*proc/self/pagemap* file, starting from version 4.0 [54].)

Another alternative is to leverage huge virtual pages that are supported by contiguous physical pages [55,60]. Since a huge page spans 2 MB of contiguous physical addresses, the attacker enables the utilization of relative offsets to access particular physical memory pages without requiring the precise translation of information from virtual to physical addresses.

**Figure 5:**
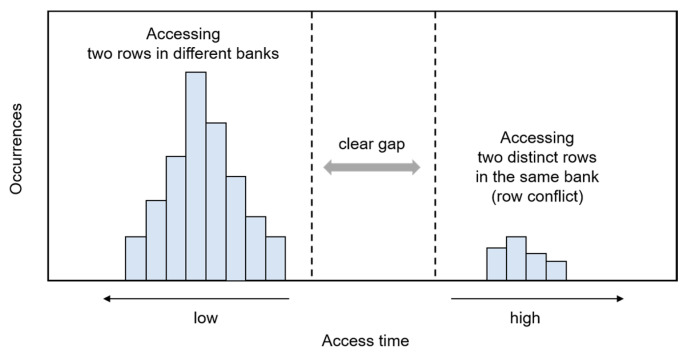
Illustrative example of latency for accessing two distinct DRAM rows.

Various methods exist for DRAM address mapping, including the utilization of DRAM access latency as a side channel, the adjustment of DRAM timing parameters, and the application of a thermal gradient on a DRAM device [55,56,57,58,59]. The authors in [55] demonstrated that accessing two distinct rows within the same bank results in a longer latency when compared to accessing two rows located in different banks, as shown in Figure 5. This latency difference stems from the row buffer’s function as a direct-mapped cache, capable of holding just a single row per bank. Consequently, this disparity can be utilized as a side channel to infer the adjacency of rows within a DRAM device.

In addition, the techniques discussed in [57,58] rely on measuring the distance from a row to the row buffer. This approach introduces errors by accessing memory using DRAM timing values that are shorter than the standard timings. In essence, data stored in a cell closer to the row buffer has a shorter distance to traverse compared to data stored farther away, reducing the likelihood of encountering errors. Hence, the likelihood of errors can be used to estimate DRAM addresses approximately.

The authors in [56] proposed a method to determine the physical positions of DRAM cells by conducting a retention error analysis while subjecting the DRAM device to a controlled temperature gradient. This allows attackers to derive the spatial relationships between individual DRAM cells, facilitating the execution of highly focused rowhammer attacks.

### 3.2. Bypassing a Cache Hierarchy

Bypassing a cache hierarchy is an essential prerequisite for achieving direct access to DRAM and subsequently executing a rowhammer attack. Existing cache bypass methods can be broadly categorized into three primary approaches: cache flushing, cache eviction, and non-temporal store-based bypassing [3,5,60,61].

Cache flushing, which involves purging data from the cache, represents the most straightforward method to guarantee that each memory access originates from DRAM instead of the CPU cache. For x86 architecture, the research [3] effectively utilized the *clflush* command, specifically designed for cache flushing. In the context of ARM architecture, such as ARMv7, the *cacheflush*() system call can be employed [60].

Researchers in [5,62,63] proposed cache eviction techniques that do not rely on *clflush* or *cacheflush()* instructions, which may be unavailable on recent architectures. These techniques involve carefully crafting memory access patterns to indirectly evict cache lines, ensuring that subsequent accesses target DRAM.

Furthermore, non-temporal store instructions, such as *movnti* or *movntdq* for x86 architecture, as demonstrated in [61], offer an alternative approach to bypassing the cache. To ensure that each non-temporal store instruction reaches the DRAM chip, write-combining buffers must be flushed. This can be achieved by following the non-temporal store instruction with cached memory access to the same address where the instruction writes data.

### 3.3. Escaping a Sandbox

JavaScript operates within a strict sandboxed environment, limiting its access to files and system services and lacking concepts like virtual addresses and pointers. Additionally, its timing precision falls short of that in native code, making rowhammer attacks seem challenging. However, pioneering research by [64,65] has revealed that JavaScript-based cache attacks can exploit timing accuracy to differentiate cache hits from misses, opening the door to timing attacks.

In JavaScript, when memory is allocated, browsers like Firefox and Google Chrome designate an anonymous 2 MB page for a large array. Accessing this array with a 4 KB address triggers page faults, causing latency spikes each time the 2 MB page commences. This distinctive behavior enables the identification of the 2 MB page frame. Armed with knowledge about the array’s offset, it is possible to gain insight into the least significant 21 bits of both virtual and physical addresses. Armed with these data, it is possible to create a tool to convert virtual addresses into their corresponding physical addresses.

### 3.4. Bypassing Target Row Refresh

Target Row Refresh (TRR) is a well-known defense mechanism against rowhammer attacks. Unlike traditional DRAM, which refreshes rows at regular intervals, TRR selectively refreshes rows identified as potential victim rows, effectively thwarting attacks. Recent strategies to bypass TRR can be broadly classified into two approaches: those that exploit weaknesses within TRR’s operation and those that completely circumvent TRR. The former includes techniques like the half-double rowhammer attack, which will be discussed in Section 4. The latter encompasses methods such as TRRespass [66] and BLACKSMITH [67], a multi-sided rowhammer strategy utilizing fuzzing techniques.

## 4. Repetitive Access Patterns for Rowhammer

There exist various row access patterns for executing rowhammer attacks, categorized into three primary types: single-sided, double-sided, and multi-sided attacks. In a single-sided rowhammer attack, repetitive memory accesses are focused on only one row, typically the one adjacent to the target row. In a double-sided rowhammer attack, two memory rows are repeatedly accessed, effectively surrounding the target row. A multi-sided rowhammer attack involves more than two memory rows to circumvent rowhammer mitigation techniques such as TRR [8,62,68].

### 4.1. Single-Sided Attack

The single-sided attack is primarily directed at a single row adjacent to the target victim row. However, when the memory controller utilizes an open-page policy, where a memory row remains buffered until the next memory row is accessed, the single-sided attack requires accessing of two separate rows within the same bank to clear the contents of the row buffer. This discrepancy arises despite the name “single-sided attack”, suggesting the targeting of only a single memory location.

In contrast to the open-page policy, modern systems have adopted more advanced memory controller policies that proactively close rows ahead of their actual necessity, aiming to enhance overall performance [69,70,71]. Building upon this shift, authors in [8] proposed a novel technique known as *one-location hammering*. In this approach, the attacker executes a *Flush + Reload* [8] loop exclusively on a single memory address, operating at the maximum possible frequency. This continuous activity effectively reopens the same DRAM row every time the memory controller attempts to close it. Since one-location hammering does not access different rows within the same bank, it has the capacity to bypass certain existing defense mechanisms designed to detect the original single-sided attack patterns.

### 4.2. Double-Sided Attack

**Figure 6:**
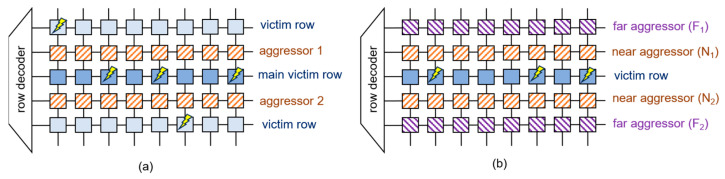
Illustrative example of (**a**) double-sided attack and (**b**) half-double attack.

The double-sided attack entails the simultaneous hammering of two memory rows, effectively sandwiching the target victim row, as illustrated in Figure 6a. In contrast to the single-sided attack, the double-sided approach typically has the potential to induce a greater number of bit flips. However, it demands a more extensive understanding of virtual-to-physical mappings, as the two rows subjected to hammering must be strategically positioned on opposite sides of the target row. The research discussed in [8,47,62] effectively leveraged the double-sided attack to induce successful bit flips via rowhammer.

### 4.3. Multi-Sided Attack

Considering that both the single-sided attack and double-sided attack are designed to induce bit flips in an adjacent row, rowhammer defense mechanisms such as TRR often operate under the assumption that aggressor–victim pairs are indeed adjacent. To evade detection, researchers have explored the concept of multi-sided attacks, exemplified by techniques such as half-double [62] and TRRespass [66].

The half-double technique initially targets two far aggressors, F_1_ and F_2_, as shown in Figure 6b. This deliberate choice ensures that only a subtle charge leakage occurs in the victim row, which is insufficient to induce a bit flip. Interestingly, the half-double approach leverages TRR counterintuitively. By consistently accessing F_1_ and F_2_ beyond the threshold that triggers a TRR, a TRR is induced in the adjacent row near aggressors N_1_ and N_2_. This action subsequently involves accessing the near aggressor rows, which, in turn, influences the victim row, resulting in a bit flip. Additionally, the blaster [63] with a row distance of 4 from the victim row is currently being researched.

TRRespass introduced a black-box multi-sided rowhammer fuzzer that discovers accessing patterns effective under TRR based on the observation that modern TRR implementations are generally susceptible to rowhammer attacks with many aggressor rows [66].

## 5. Exploitation of Rowhammer

Rowhammer attacks possess the potential to undermine the fundamental pillars of computer system security, which are commonly represented by the confidentiality, integrity, and availability (CIA) triad. Table 2 presents a list of referenced papers pertaining to the CIA triad in the context of rowhammer attacks.

### 5.1. Confidentiality Degradation

Rowhammer attacks targeting an operating system have the potential to facilitate privilege escalation, thereby degrading confidentiality. In the study conducted by Seaborn et al. [3], researchers demonstrated the exploitation of rowhammer-induced bit flips to attain kernel privileges on x86-64 Linux systems, even when executed as an unprivileged userland process. On systems vulnerable to the rowhammer issue, this process was able to initiate bit flips within page table entries (PTEs). Consequently, it successfully acquired the capability to modify its own page table, and thus granting itself read-write access to the entirety of physical memory.

Moreover, rowhammer attacks can target shared memory resources in cloud computing environments where multiple virtual machines (VMs) share the same physical hardware. In the study conducted by Xiao et al. [5], a privilege escalation attack was executed within a cloud environment, enabling malicious users to acquire read and write permissions to other users’ VMs. The attack method involved identifying weak memory DRAM cells via memory profiling and employing double-sided rowhammering. Subsequently, the attack mapped the page directory within the VM’s operating system kernel to the page containing the weak memory cell. The attack then executed the rowhammer technique to flip the vulnerable bits within the page directory at the anticipated locations, redirecting them to a different page table than originally intended. Via this process, the researchers demonstrated that a guest VM can read and write any memory page on the machine.

In addition, rowhammer attacks can target the memory-storing cryptographic keys. If an attacker successfully flips bits in the memory where encryption keys are stored, they can decrypt sensitive data, compromising the confidentiality of encrypted communications or stored data. In the study conducted by Kaveh Razavi et al. [4], the authors demonstrated how rowhammer attacks could compromise the security of OpenSSH public-key authentication and forge GNU Privacy Guard signatures from trusted keys. This compromise, in turn, threatened the integrity of the Ubuntu/Debian update mechanism. Also, the study conducted by Andrew Kwong et al. [2] revealed the alarming possibility of extracting an RSA-2048 key on OpenSSH 7.9 via a combination of their proposed memory profiling and double-sided rowhammering techniques.

### 5.2. Integrity Degradation

Rowhammer attacks can alter the content of a DRAM cell without the need for direct access, thereby leading to a deterioration of data integrity. The study presented in Rowhammer.js [6] introduced the rowhammer attack framework that necessitates nothing more than a website utilizing JavaScript to induce errors within a remote computing system. Furthermore, the study in ECCploit [7] demonstrated integrity degradation within error correction code (ECC) memory. This research involves identifying bit flips that ECC can initially correct, only to subsequently combine these bit flips in such a way that ECC becomes incapable of correction or detection.

**Figure 7:**
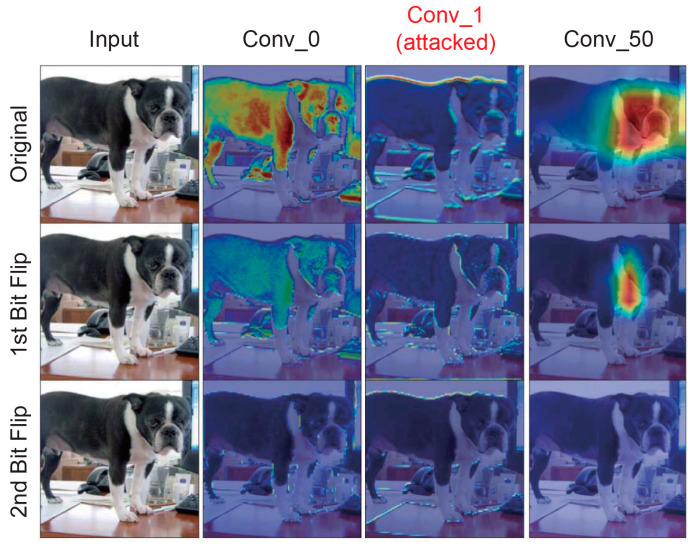
Change in the attention map by a couple of bit flips in MobileNetV2 [73].

Additional studies cited in [72,73] have demonstrated that rowhammer-induced bit flips within the neural network parameter bits stored in DRAM can significantly undermine inference accuracy. The study in [72] showed that only 13 bit flips of weight parameters of a ResNet-18 convolutional neural network could degrade top-1 accuracy from 69.8% to 0.1%. The study in [73] underscored that a mere couple of bit flips within a mobile-friendly neural network can notably impair its accuracy. In Figure 7, attention maps at various convolution layers in MobileNetV2, extracted using Grad-CAM [74], reveal a substantial shift in the map’s location after just two bit flips.

### 5.3. Availability Degradation

It is possible to conduct a denial-of-service attack in a cloud environment using rowhammer attack methods. Such an attack has the potential to reduce accessibility in the cloud. Intel Software Guard Extensions (SGX) are x86 instruction extensions that verify the OS, hypervisor, and hardware for tampering. If there are any errors in confidentiality or integrity, Intel SGX is suspended until the system is restarted. These techniques can be misused to shut down numerous cloud systems by introducing errors into several machines [8,9].

## 6. Rowhammer Defenses

Despite persistent and ongoing research efforts to develop defense strategies against rowhammer attacks, vulnerabilities remain prevalent [2,3,4,5,6,7,8,9]. The diminishing technology nodes in DRAM chips amplify the threat, enabling rowhammer attacks to succeed with fewer row activations [46,47,68]. This underscores the need to reevaluate and enhance existing defense mechanisms. In this section, we categorize rowhammer defense strategies into three areas based on where their mitigation technique is applied: software, memory controller, and DRAM as shown in Table 3. Subsequently, we analyze strategies within each category using the criteria of protection concepts, tracking mechanisms, and remedies.

Protection concepts are divided into two types, deterministic and probabilistic, based on the degree of protection they offer against rowhammer attacks. Deterministic methods are designed to entirely prevent rowhammer attacks under specific environments and conditions. They employ distinct rules or mechanisms, such as using a counter to monitor the number of ACTIVATE commands in a row and refreshing that row before a predetermined threshold is reached. In contrast, probabilistic methods aim to thwart rowhammer attacks with a certain likelihood, thereby minimizing performance overhead. While they do not guarantee the complete prevention of rowhammer attacks, their goal is to increase the complexity of the attack, making it more challenging for the attacker.

The tracking mechanism involves various proposed mechanisms to track attacks, with counters being the most commonly used method. Using counters allows for the precise monitoring of the number of ACTIVATE commands in a row within DRAM [11,12,13,14,15,16,17,20,21,22]. By refreshing the specific row before it exceeds a predetermined threshold, attacks can be prevented. Additionally, other mechanisms, such as Queue or Cache, have been suggested to track and detect attack patterns [18,23,24,25,26].

Remedy refers to the countermeasures implemented after detecting a rowhammer attack to mitigate or defend against it. The focus is on minimizing the impact of the attack or completely blocking it. For instance, the reactive refresh method refreshes adjacent rows upon detecting an attack to prevent bit flips. Proactive throttling [1,16,75] delays the activation frequency of DRAM for a certain period once an attack is detected, reducing the likelihood of a successful rowhammer attack. Another method, physical isolation [23,27,28,29,30], protects sensitive data by physically separating them from potential attackers.

### 6.1. Software-Based Mitigations

Software-based mitigation strategies are predominantly implemented within the operating system kernel, given the operating system’s direct oversight of hardware resources. These strategies can be broadly categorized into two types: heuristic-based attack detection [10,11,36] and physical isolation [27,28,29,30].

#### 6.1.1. Heuristic-Based Attack Detection

Heuristic-based attack detection leverages hardware performance counters to identify potential attackers and refresh the rows at risk of being bit-flipped before any damage occurs. For instance, ANVIL [10] employs CPU performance counters to gather memory access data and monitor DRAM rows. By observing the DRAM row access patterns in the cache, it can force a refresh on neighboring rows that might be victimized if repeated accesses are detected. On the other hand, SoftTRR [11] offers deterministic protection to the page table. It does so by monitoring memory accesses to all rows near the page table using a counter. When the number of observed accesses surpasses a set threshold, it refreshes the rows. This strategy is particularly effective against page table-based privilege escalation attacks, which rank among the most detrimental system attacks. However, a notable limitation is that hardware performance counters are not universally available across all CPUs. This means rowhammer attacks might still transpire on devices that are not being monitored.

RADAR [36] focuses on detecting rowhammer triggers by concentrating on the abnormal electromagnetic (EM) signals emitted during the hammering process. It uses a wireless-based external device to capture the spectrum of the DRAM clock signal and employs a convolutional neural network (CNN) to detect anomalous patterns.

#### 6.1.2. Physical Isolation

**Figure 8:**
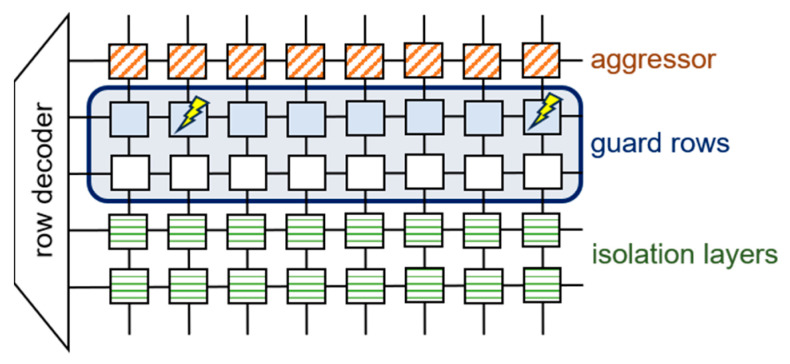
Illustrative example of physical isolation.

Physical isolation has garnered substantial attention as a robust software-implemented defense against rowhammer attacks. This technique employs guard rows to absorb bit flips, thereby limiting an attacker’s ability to manipulate bits. As illustrated in Figure 8, even when a bit flip transpires, the guard rows absorb the impact, ensuring the attacker’s influence does not permeate the isolation layer. CATT [23] introduces a protective layer for the kernel by placing guard rows between it and the user memory. By confining the attacker’s reach to the user space, it ensures that bit flips induced by rowhammer remain within the attacker’s domain, preserving kernel stability. However, a study by Gruss et al. [8]. Revealed that attacks could be executed irrespective of the isolation between user and kernel memory. Addressing this, RIP-RH [27] augmented Linux’s page allocation mechanism, enabling the dynamic management of simultaneous user-space processes. By physically segregating each process, it showcased the inability of attacks on adjacent memory segments.

ZebRAM [30] employs a zebra pattern to isolate rows that house critical data. But, dedicating 50% of memory to guard rows is inefficient. To counteract this, it repurposes the guard rows as optionally compressed swap spaces, enhancing performance. Both GuardION and ALIS adopt a targeted approach to memory protection, emphasizing specific attack vectors rather than blanket protection. GuardION [29] zeroes in on Direct Memory Access (DMA)-based attacks, a primary threat vector for mobile devices, while ALIS [28] hones in on remote attacks that pinpoint memory allocated to DMA buffers.

The challenge with physical isolation techniques is the inevitable reduction in available memory capacity. As technology nodes in DRAM chips continue to shrink, the memory area resilient to rowhammer attacks diminishes [1,47]. The expanding scope of rowhammer vulnerabilities necessitates a more extensive physical gap between sensitive data and the memory regions accessible to potential attackers [76].

There are also mitigation techniques that reinforce page table isolation. All traditional kernel privilege escalation attacks aim to corrupt the page table. In response, Cell-Type-Aware memory allocation (CTA) [37] proposes a memory allocation technique at the operating system (OS) level that assigns page table pages to a dedicated memory area, which is physically high-addressed and separated by guard rows. Assigning page table entries (PTEs) to high physical addresses means that even if a bit-flip attack changes the address to point to a lower new physical address, it will not point to another PTE, thus preventing kernel privilege escalation. PT-Guard [19] protects the page table from tampering by storing a message authentication code (MAC) within the Page Table Entry (PTE) cache line itself to detect data tampering.

Copy-on-Flip [39] enhances ECC at the software level to mitigate attacks, a method previously considered insufficient for defending against rowhammer. As soon as an attacker successfully templates enough bit flips, the vulnerable victim page is taken offline, and at the same time, the affected data is protected via migration.

Software-based solutions, while innovative, pose practical deployment challenges and frequently come with significant performance overheads. All isolation methods have a limited scope of target achievement and require the application of DRAM-aware memory allocation, making their adoption in commercial systems challenging. Additionally, these methods often rely on the incorrect assumption that logical and physical DRAM addresses are identical or they are customized to address specific attack scenarios, limiting their overall efficiency. Recognizing these constraints, the focus of research has shifted toward solutions within memory controllers and DRAM since 2019.

### 6.2. Memory Controller-Based Mitigations

**Figure 9:**
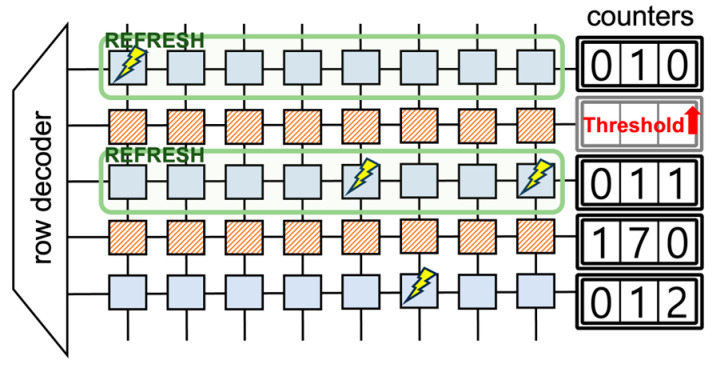
Illustrative example of counter-based mitigation.

The predominant defense strategy employed by memory controllers involves monitoring the activation commands of DRAM using a counter. When this counter surpasses a set threshold, it identifies a potential rowhammer attack and refreshes neighboring rows to avert bit flips, as shown in Figure 9. While this approach offers robust protection deterministically, the memory overhead associated with the counter is substantial. Consequently, a pivotal challenge in counter-based defense mechanisms is minimizing the counter’s overhead.

To address the overhead, counter-based defense mechanisms use different counter-structures. Counter-based Row Activation (CRA [1,24]) method suggests caching the counters for recently accessed rows within the memory controller and relegating the remainder to the main memory. This approach stems from the prohibitive cost of maintaining an individual counter for every row directly within the memory controller. Another method, the Counter-based Tree (CBT [13]), segments rows into clusters to monitor their activations. It dynamically recalibrates the range of rows each counter oversees based on the activation frequency of rows. By accounting for the frequency of row accesses, CBT enhances the energy and spatial efficiency of the counters. Nonetheless, both the CRA and CBT techniques experience performance downturns under antagonistic memory access patterns. This decline is attributed to frequent counter cache misses and recurrent refreshes when multiple rows fall under the purview of a singular counter [14,77].

To address the performance setbacks, several alternative strategies have been put forward. Time Window Counter (TWiCe) [14] identifies rowhammer attacks using a minimal set of counters by periodically removing rows with insufficient activation frequency. This technique employs the lossy-counting algorithm to evaluate the greater row activation frequency and DRAM cell retention time and determines the maximum number of counter entries required per DRAM bank. An extension of TWiCe was introduced in CTA [37]. Graphene [15] employs the Misra-Gries algorithm [78] to accurately pinpoint and monitor rows activated frequently. This counter-based probabilistic method offers assured protection at a reduced expense. BlockHammer [16] identifies attacks by assessing the resemblance between a specific thread’s memory access pattern and a genuine rowhammer attack. Instead of preemptively refreshing potential victim row, it provides a proactive throttling technique that actively limits memory accesses that are deemed malicious. Utilizing two Counting Bloom Filters (CBFs), it evaluates the activation frequency of all rows and blacklists those exceeding a predetermined threshold, thereby preventing further access to detected attackers. HammerFilter [40] similarly isolates attackers probabilistically. It optimizes Counting Bloom Filter (CBF) operations by adding a HALF-DELETE operation to reduce the access frequency of refreshed rows.

Beyond counter-centric defenses, both probabilistic and physical isolation solutions have been explored. The probabilistic method, Probabilistic Adjacent Row Activation (PARA [1]), activates surrounding rows of a suspected attack probabilistically upon detecting an access pattern. By probabilistically activating rows, it diminishes performance and energy overheads, making the system less predictable to adversaries and thereby decreasing rowhammer-induced error likelihood [70]. While it does not entirely thwart rowhammer attacks, it curtails the attack’s efficacy via irregular refresh patterns, thereby mitigating potential damage. Discreet-PARA [41] significantly reduces the performance overhead of PARA by combining a counter that counts the activities in a section of a bank with cache storage space, triggering the original PARA only when activities occur in frequently activated sections. ProHIT [26] and MRLoc [18] recognize the limitations of PARA and enhance performance by adding memory accesses to track the history of row activations and issue additional refreshes. ProHIT [26] manages access history by randomly adding neighboring rows of activated rows to a priority table. By checking the table at each refresh

And performing an additional refresh on the row with the highest priority, ProHIT prevents damage to the most likely victims, thereby increasing the reliability of PARA. MRLoc [18] solves the high-power consumption problem that can occur with PRoHIT. It stores neighboring rows of activated rows in a queue, uses the frequency of insertion into the queue to determine the refresh probability, and then uses this probability to provide optimized additional refreshes.

**Figure 10:**
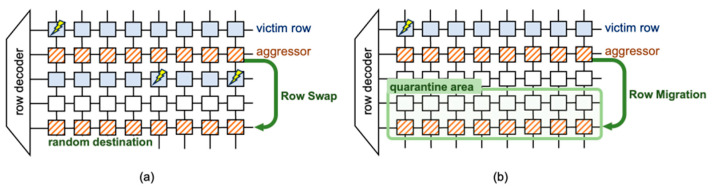
Illustrative concept of (**a**) RRS [31] and (**b**) Aqua [32].

Randomized Row Swap (RRS) [31] and Aqua [32] adopt Physical Isolation as their countermeasure, severing the spatial link between attacker and victim rows to stave off rowhammer attacks. RRS [31] identifies frequently activated rows via a streaming algorithm using the Hot-Row Tracker (HRT) and Row-Indirection Table (RIT) and then isolates these attacker rows by replacing them with randomly selected rows, protecting potential victims as shown in Figure 10. SRS [38] discovered a vulnerability in RRS caused by latent row activations resulting from swap–unswap operations and introduced Secure Row-Swap to counter this issue. Aqua [32] dynamically migrates attacker rows to the quarantine area, disrupting the spatial relationship between attacker and victim rows, as illustrated in Figure 10b. While these strategies considerably diminish rowhammer attack susceptibility, they come with performance and memory overhead trade-offs.

An alternative Memory Controller-Based Mitigation method is LightRoAD [42]. LightRoAD leverages hardware counters to monitor cache misses, cache flushes, and DMA accesses, triggering responsive actions when the cumulative values of these counters reach a predefined threshold. This approach enables the system to proactively detect and respond to manipulative actions, providing insights into the specific components and processes that may be exploiting vulnerabilities.

Defense mechanisms rooted in memory controllers boast the merit of being largely executable without necessitating hardware modifications. These approaches have the strength of directly identifying DRAM access patterns. However, they do not provide absolute protection as they overlook the physical proximity within the DRAM chip [79]. Most data integrity check-based solutions only have detection capabilities and do not include correction features. PRoHit [26] and MRLoc [18] have significantly optimized PARA [1], but they are still vulnerable to certain attacks [15]. The augmented area required for the counter structure and the heightened expense associated with modifying the memory controller also pose challenges.

### 6.3. DRAM-Based Mitigations

Early hardware-centric defenses against rowhammer, as proposed by Kim et al. [1], encompassed strategies like elevating the refresh rate, implementing target row refresh (TRR [12,13,14,15,16,18,20,23,28,29,30,31]), and utilizing error-correcting codes (ECC [7,76]). By amplifying the DRAM’s refresh rate, the window of opportunity for an attacker to execute a rowhammer assault narrows, potentially diminishing the attack’s potency [80,81,82]. However, this method inadvertently escalates power consumption and hampers system performance. With the advent of DDR4, a more sophisticated defense mechanism, TRR [12,13,14,15,16,18,20,23,28,29,30,31], was ushered in. TRR leverages hardware counters to scrutinize memory access behaviors. Instead of the conventional blanket refresh approach applied to all memory, TRR selectively refreshes specific DRAM rows perceived to be susceptible to rowhammer. This targeted approach can curtail issues related to power consumption and performance degradation. TRR can be orchestrated in software, within the CPU’s memory controller, or directly inside the DRAM. Nonetheless, relying solely on TRR is not foolproof against certain intricate attack modalities [66]. ECC [7,76], while designed to rectify errors, remains susceptible to rowhammer onslaughts, especially those that manipulate multiple bits within a memory word. Kim et al. highlighted this vulnerability. Although employing ECC can ramp up the intricacy of the attack, it does not offer an absolute safeguard. This limitation was evident in attacks like ECCploit [7].

Recent research has aimed to diminish the performance overhead associated with conventional DRAM-based mitigation techniques. ProTRR [17] introduced a method leveraging FEINTING technology to proactively identify and refresh rows potentially targeted by attacks. Being probabilistic, it zeroes in on the most frequently attacked rows, although some might still evade detection. This offers a more balanced trade-off among DRAM vulnerability, counter quantity, and additional refreshes compared to older methods. REGA [33] combats rowhammer by concurrently refreshing distinct rows during data transfer. It separates the DRAM sense amplifier’s row refresh operation from the data transfer task. By sequentially refreshing all rows in the DRAM sub-array receiving the activation command, it obviates the need to monitor the attacker. This approach is pivotal for future mitigation techniques as it scales the refresh count based on activation intensity. HiRA [34] can concurrently refresh DRAM rows while activating or refreshing other rows within the same bank. This minimizes performance degradation from periodic refreshes by cutting down the overall row refresh latency.

DRAM-based solutions predominantly revolve around additional preventive refreshes for potential rowhammer victim rows [17,21,22,25,33,34,35]. However, securing adequate non-disruptive time at the DRAM interface for these refreshes is challenging. Techniques like invoking an adjacent row refresh request (ARR) or incorporating a refresh process should be explored to ensure potential victim rows have adequate time for rowhammer protective measures. Mithril [22] tackled this challenge by synergizing rowhammer defense efforts between the memory controller and DRAM. It is anchored in the Refresh Management (RFM) introduced in the DDR5 standard [83]. Here, the memory controller dispatches an RFM to the target bank at a specific activation frequency without pinpointing the target row. The DRAM then harnesses the time buffer provided by the RFM command to implement suitable rowhammer protective actions. A counter-based streaming algorithm determines the rows needing a refresh, and a greedy selection strategy guarantees deterministic protection. Panopticon [25] adapts an existing DDR4 specification signal, ALERTn, to deter the memory controller from initiating a new DRAM command when a potential victim row requires refreshing. This utilizes unique counters for DRAM rows and, when a counter reaches the rowhammer threshold, temporarily queues the row address, masquerading as a missed access to delay other accesses. The Silver Bullet Technique [43] analyzes the worst-case access pattern, defines the tolerable maximum hammering value, and then proactively refreshes potential victim rows.

## 7. Discussion: Rowhammer on DDR5 DRAM

Previous studies have focused on the execution of rowhammer attacks as well as the development of mitigations for DDR3 or DDR4 memory modules [1,2,3,4,5,6,7,8,9,10,11,12,13,14,15,16,17,18,19,20,21,22,23,24,25,26,27,28,29,30,31,32,33,34,35,36,37,38,39,40,41,42,43,44,45,46,47,48,49,54,55,56,57,58,59,60,61,62,63,64,65,66,67,68,69,70,71,72,75,76,77,78,79,80,81,82]. However, there exists a need to expand this research to the latest generation, such as DDR5. DDR5 DRAM undergoes a more aggressive scaling process, aiming for increased density and larger bandwidth [84,85,86,87]. The increased scaling of the DRAM cell in DDR5 renders it more susceptible to data retention failures. In response to this vulnerability, the DDR5 standard incorporates a Single-Error-Correction (SEC) code as an In-DRAM error correction code, enhancing on-chip reliability [85,87].

**Figure 11:**
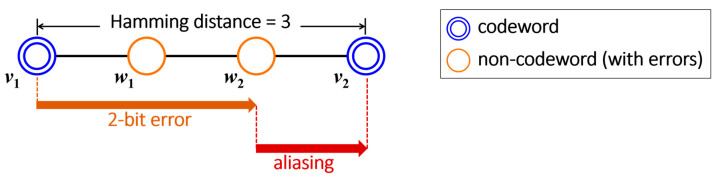
Aliasing on two-bit error occurrence by In-DRAM ECC.

In scenarios involving SEC-protected data, a two-bit error induced by phenomena like rowhammer may be erroneously transformed into a triple-bit error due to incorrect decoding [85]. SEC codes guarantee a minimum Hamming distance of three between valid codewords. Illustrated in Figure 11, consider codewords *v*_1_ and *v*_2_ with a Hamming distance of three. If a two-bit error occurs on *v*_1_, resulting in *w*_2_, and the Hamming distance between *w*_2_ and *v*_2_ is one, the SEC decoder inaccurately interprets the received word *w*_2_ as *v*_2_. Consequently, the number of error bits in a DRAM chip increases from two to three. Also, the aliasing has the potential to disrupt the consistent asymmetric DRAM error pattern caused by rowhammer, which typically induces 1-to-0 (0-to-1) errors in true cells (anti-cells) [1,2,37].

It is noteworthy that established mitigation techniques such as CTA [37] have relied on the predictable asymmetric DRAM error pattern induced by rowhammer, a reliance that may become obsolete in the presence of In-DRAM error correction code. Hence, future research on DDR5 rowhammer needs to identify the aliasing issue stemming from the In-DRAM error correction code feature. To address the aliasing issue in DDR5 DRAM, future research should delve into developing effective countermeasures and mitigations. Understanding and mitigating the impact of rowhammer-induced errors, especially in scenarios involving SEC-protected data, is crucial for maintaining the integrity and reliability of DDR5 memory modules.

## 8. Conclusions

In this paper, we provided a comprehensive review of recent advancements in rowhammer attacks, breaking down the attack process into three stages: setup, repetitive access, and exploitation. We delved into existing techniques within each stage, offering insights into their underlying mechanisms. Furthermore, we conducted an exhaustive examination of defense strategies against rowhammer attacks, categorizing them into three distinct groups based on where each mitigation technique is applied.

## Figures and Tables

**Table 1:** List of referenced papers for rowhammer setup.

Setup	Cited Paper	Year	Methods
Memory profiling	Project Zero [3]	2015	Pagemap/Huge pages/Timing
	Pagemap [54]	2015	Pagemap
	DRAMA [55]	2015	Pagemap/Huge pages/Timing
	Cross-VM Row Hammer [5]	2015	Timing
	Rambleed [2]	2020	Pagemap
	Row hammer with crosshair [56]	2016	The retention error behavior with respect to the temperature
	Solar-DRAM [57]	2018	Timing
	Design-induced latency variation [58]	2017	Timing
	A surgical precision hammer [59]	2018	Pagemap/Huge pages/Timing
	Drammer [60]	2016	Pagemap/Huge pages/Timing
Bypassing a cache hierarchy	DRAM disturbance errors [1]	2014	Cache Flushing
	A new approach [61]	2016	Non-temporal store-based bypassing
	Half-Double [62]	2022	Cache Eviction
	BLASTER [63]	2023	Cache Eviction
Bypassing mitigation techniques	Memory deduplication attacks in sandboxed JavaScript [64]	2015	Escaping a Sandbox
	The spy in the sandbox [65]	2015	Escaping a Sandbox
	Rowhammer.js [6]	2016	Escaping a Sandbox
	TRRespass [66]	2020	Bypassing TRR
	BLACKSMITH [67]	2022	Bypassing TRR

**Table 2:** List of referenced papers pertaining to the CIA triad.

CIA Triad	Cited Paper	Year	Target
Confidentiality degradation	Project Zero [3]	2015	An operating system for privilege escalation
	Drammer [60]	2016	An ARM-based device for privilege escalation
	Cross-VM Row Hammer [5]	2016	The physical hardware in virtual machines (VMs)
	Flip Feng Shui [4]	2016	The memory-storing cryptographic keys
	RAMBleed [2]	2020	An operating system for privilege escalation
Integrity degradation	Cross-VM Row Hammer [5]	2016	Withing an OpenSSH server
	Rowhammer.js [6]	2016	Within a remote computing system
	ECCploit [7]	2019	Within error correction code (ECC) memory
	Bit-flip attack [72]	2019	Within the neural network, parameter bits stored in DRAM
	ZeBRA [73]	2021	Within the neural network, parameter bits stored in DRAM
Availability degradation	SGX-Bomb [9]	2017	Accessibility in the cloud
	Another Flip [8]	2018	Accessibility in the cloud

**Table 3:** Overview of recent rowhammer mitigations.

Location	Mechanism	Year	Protection Concepts	Tracking Mechanism	Remedy
Software	COF [39]	2023	Deterministic	Counter	Physical Isolation
	SoftTRR [11]	2021	Deterministic	Counter	Reactive Refresh
	RADAR [36]	2020	Deterministic	-	Reactive Refresh
	RIP-RH [27]	2019	Deterministic	-	Physical Isolation
	CTA [37]	2019	Deterministic	-	Physical Isolation
	ALIS [28]	2018	Deterministic	-	Physical Isolation
	GuardION [29]	2018	Deterministic	-	Physical Isolation
	ZebRAM [30]	2018	Deterministic	-	Physical Isolation
	CATT [23]	2017	Deterministic	Cache	Physical Isolation
	ANVIL [10]	2016	Probabilistic	Counter	Reactive Refresh
Memory Controller	LightRoAD [42]	2023	Deterministic	Counter, Cache	Reactive Refresh
	SRS [38]	2023	-	Counter	Reactive Refresh
	PT-Guard [19]	2023			
	Hydra [12]	2022	-	Counter	Reactive Refresh
	Aqua [32]	2022	Probabilistic	-	Physical Isolation
	RSS [31]	2022	Probabilistic	-	Physical Isolation
	Discreet-PARA [41]	2021	Probabilistic	Counter	Reactive Refresh
	BlockHammer [16]	2021	Deterministic	Counter	Proactive Throttling
	HammerFilter [40]	2021	Deterministic	Counter	Proactive Throttling
	CAT-TWO [20]	2020	Deterministic	Counter	Reactive Refresh
	Graphene [15]	2020	Deterministic	Counter	Reactive Refresh
	TWiCe [14]	2019	Deterministic	Counter	Reactive Refresh
	MRLoc [18]	2019	Probabilistic	Counter, Queue	Reactive Refresh
	CBT [13]	2016	Probabilistic	Counter	Reactive Refresh
	CRA [24]	2015	Probabilistic	Counter, Cache	Reactive Refresh
	PARA [1]	2014	Probabilistic	-	Increased Refresh Rate
DRAM	REGA [33]	2023	Deterministic	-	Reactive Refresh
	Dsac [21]	2023	Probabilistic	Counter	Reactive Refresh
	CSI [35]	2023	-	-	-
	HiRA [34]	2022	Probabilistic	-	Reactive Refresh
	Mithril [22]	2022	Deterministic	Counter	Reactive Refresh
	ProTRR [17]	2022	Probabilistic	Counter	Reactive Refresh
	Silver Bullet Technique [43]	2021	Probabilistic	-	Reactive Refresh
	Panopticon [25]	2021	Deterministic	Counter, Queue	Reactive Refresh
	ProHIT [26]	2017	Probabilistic	Queue	-

## Data Availability

All data that support the findings of this study are included within the article.
